# Monitoring and external control of pH in microfluidic droplets during microbial culturing

**DOI:** 10.1186/s12934-020-1282-y

**Published:** 2020-01-29

**Authors:** Miguel Tovar, Lisa Mahler, Stefanie Buchheim, Martin Roth, Miriam A. Rosenbaum

**Affiliations:** 1 Bio Pilot Plant, Leibniz Institute for Natural Product Research and Infection Biology, Hans Knöll Institute, 07745 Jena, Germany; 2 Faculty of Biology and Pharmacy, Friedrich Schiller University, 07743 Jena, Germany

**Keywords:** Droplet microfluidics, pH regulation, Inter-droplet transport, *E. coli* K12- and B-strain, Miniaturized fermentation

## Abstract

**Background:**

Cell-based experimentation in microfluidic droplets is becoming increasingly popular among biotechnologists and microbiologists, since inherent characteristics of droplets allow high throughput at low cost and space investment. The range of applications for droplet assays is expanding from single cell analysis toward complex cell–cell incubation and interaction studies. As a result of cellular metabolism in these setups, relevant physicochemical alterations frequently occur before functional assays are conducted. However, to use droplets as truly miniaturized bioreactors, parameters like pH and oxygen availability should be controlled similar to large-scale fermentation to ensure reliable research.

**Results:**

Here, we introduce a comprehensive strategy to monitor and control pH for large droplet populations during long-term incubation. We show the correlation of fluorescence intensity of 6-carboxyfluorescein and pH in single droplets and entire droplet populations. By taking advantage of inter-droplet transport of pH-mediating molecules, the average pH value of several million droplets is simultaneously adjusted in an a priori defined direction. To demonstrate the need of pH control in practice, we compared the fermentation profiles of two *E. coli* strains, a K12-strain and a B-strain, in unbuffered medium with 5 g/L glucose for standard 1 L bioreactors and 180 pL droplets. In both fermentation formats, the commonly used B-strain *E. coli* BL21 is able to consume glucose until depletion and prevent a pH drop, while the growth of the K12-strain *E. coli* MG1655 is soon inhibited by a low pH caused by its own high acetate production. By regulating the pH during fermentation in droplets with our suggested strategy, we were able to prevent the growth arrest of *E. coli* MG1655 and obtained an equally high biomass yield as with *E. coli* BL21.

**Conclusion:**

We demonstrated a comparable success of pH monitoring and regulation for fermentations in 1 L scale and 180 pL scale for two *E. coli* strains. This strategy has the potential to improve cell-based experiments for various microbial systems in microfluidic droplets and opens the possibility for new functional assay designs.

## Background

In the past decades, microbiological experimentation experienced a quantum leap due to the development of various novel molecular and analytical techniques. As a secondary consequence, these advances have deviated the attention away from classic aspects of microbiology, particularly thorough control of cultivation conditions [1]. Nevertheless, the fundamental principles and craftsmanship that have been already established for lab scale microbial cultivation should be considered for any novel experimental method development, including miniaturized cell cultivation and single cell studies [1].

Among innovative experimental strategies, droplet microfluidics has the potential to revolutionize microbiological experimentation [2], enabling ultra-high throughput of single cell or micro cultivation experiments, as well as of genomic and metabolomic approaches. Yet, two key parameters that must be monitored and controlled for robust microbiological investigations, even in picoliter droplets, are oxygen availability and pH. In particular, pH changes can indicate different metabolic compounds produced and drastically alter the cell phenotype, potentially affecting further growth or perturbing extracellular reactions. Usually pH and oxygen concentration are thoroughly monitored and controlled during standard culturing. Stirring and aeration are used to control oxygen availability, while buffers and feedback loops are widely applied to adjust pH during cultivation in automated bioreactors. However, monitoring and controlling these variables is more complex at small scales. Due to the highly reduced volume of microfluidic droplets (from pL to µL), the challenge of sensor miniaturization arises. Furthermore, such sensors must be inert and biocompatible, and provide sensitive and non-destructive measurements at such minute scale.

Using oxygen sensitive nanoparticles, we demonstrated in previous work how to monitor oxygen supply for millions of droplets simultaneously [3]. Moreover, we developed a simple strategy for active oxygenation by flushing oil through the droplet population, which results in enhanced and homogeneous oxygen availability during incubation. The improved oxygenation, for the first time, provided a microbial cultivation platform in the picoliter scale that resulted in similar growth and production yields as observed in shaking flask cultures, bridging classic microbiological craftsmanship to microfluidic droplets.

Beyond this, a strategy for pH monitoring and control is also of high importance for microbiological experimentation in droplets. Here, we endeavor to expand our strategy to include the detection and control of pH in millions of droplets simultaneously.

## Results and discussion

### pH monitoring with 6-carboxyfluorescein in single droplets and populations

**Fig. 1:**
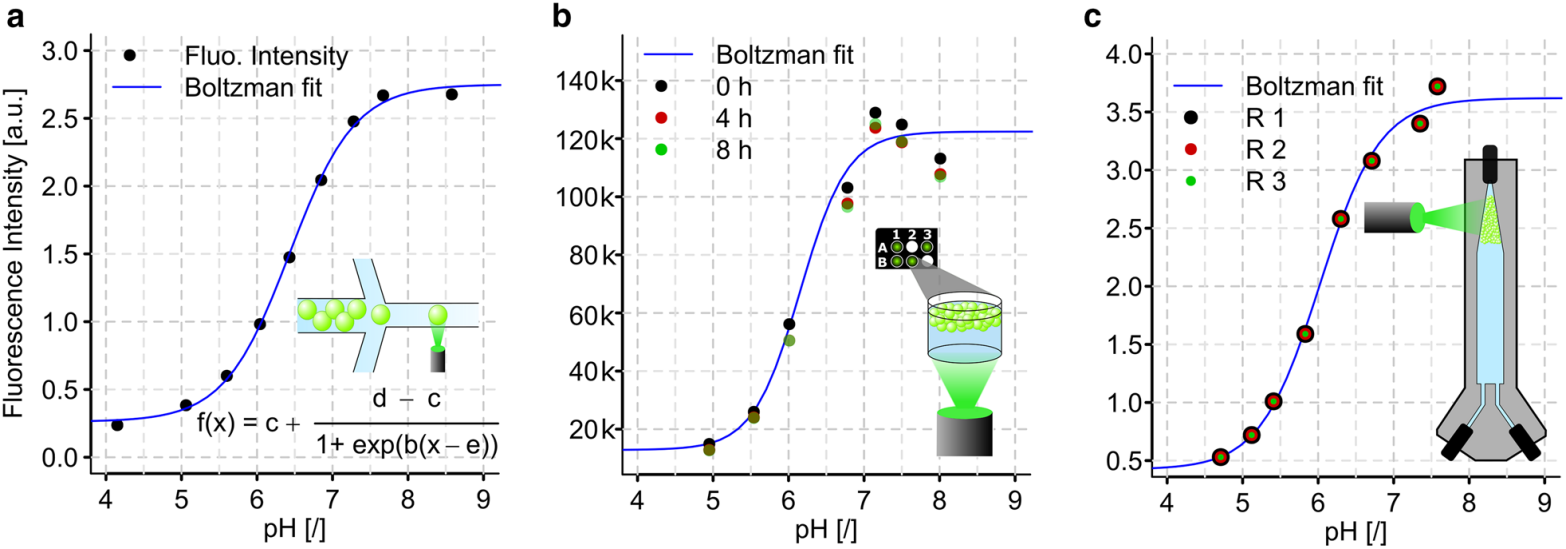
Correlation of fluorescence intensity of 6-carboxyfluorescein with pH in microfluidic droplets. **a** Fluorescence intensity of 6-carboxyfluorescein measured in droplets in flow on a microfluidic chip. pH of the bulk aqueous phase before droplet generation was determined by a conventional pH electrode. The arithmetic means of min. 1262 droplets per pH level as black points are depicted. A four parameter logistic model (Boltzman model) described by the equation in the graph was fitted to the data and is displayed as blue line. **b** Fluorescence intensity of 6-carboxyfluorescein measured for entire droplet populations of different pH in a plate reader. The intensity values for the droplets populations measured at 3 different time points are depicted, to show the variability introduced by long term incubation. A four parameter logistic model was fitted to the data of time 0 h. **c** Fluorescence intensity of 6-carboxyfluorescein measured for entire droplet populations of different pH during dynamic droplet incubation. Replicates (R) were taken from the same droplet populations after different incubation times and speeds

Our method for pH estimation is based on the dependency of the fluorescence intensity of dyes on the pH. In previous studies, estimation of the pH in single droplets using sensitive fluorescent dyes has been performed to detect the production of lactate by single cells [4–6]. The usage of 6-carboxyfluorescein dissolved in the aqueous phase at a concentration of 2 µg/mL provides a facile method for pH estimation as demonstrated with a calibration curve of droplet fluorescence intensity obtained at different pH values of the bulk aqueous phase (pH was adjusted with HCl or NaOH). As expected, the correlation of fluorescence intensity and pH value follows a sigmoidal dose response curve around the pKa of 6-carboxyfluorescein, enabling monitoring of pH in the range of 5.5–7.5 (Fig. 1a). The fluorescence intensity was determined for single droplets in flow by focusing a 488 nm laser on the microfluidic channel and detecting the emitted signal with a photomultiplier tube (PMT), resolving the approximate pH value for single droplets. To monitor the average pH value of an entire droplet population periodically, one option is to transfer the emulsion into an appropriate microtiter plate for measuring the average fluorescence intensity in a plate reader (Fig. 1b) for a fast implementation approach. As second option, the dynamic droplet incubation setup [3] can be augmented with an excitation source and a detection unit for fluorescence light allowing controlled incubation of droplets regarding oxygen supply and pH with facile connection to reinjection of the droplet population for downstream unit operations in microfluidic chips (Fig. 1c).

For all measurement options, the fluorescent signal is subject to interference caused by media components and variability during long-term experimentation due to the photo-instability of the dye. Hence, we optimized the measurement parameters like intensity of excitation light and length of measurement intervals in order to minimize fluorescence bleaching.

### Inter-droplet transport of pH mediating molecules

**Fig. 2:**
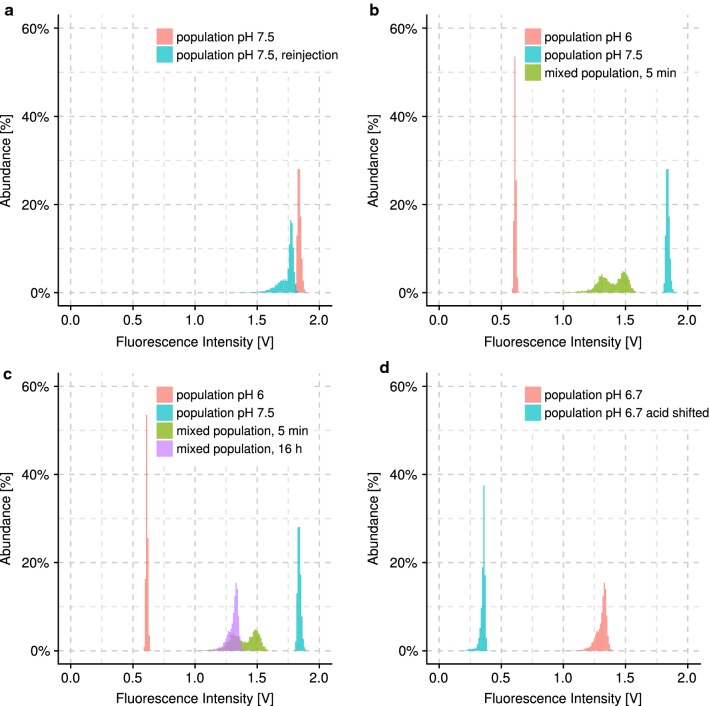
Fluorescence intensity of 6-carboxyfluorescein in dependency to pH measured for single droplets in flow, indicating the inter-droplet transport of pH mediating molecules between droplet populations. Histograms depict the abundance distribution of fluorescence intensity per single droplet for droplet populations (bin width 0.01 V). The abundance was normalized to the total count for each population. A minimum of 2880 droplets was analyzed per population. **a** Abundance distribution for a droplet population of pH 7.5 during generation and during immediate reinjection to a microfluidic chip. The variation between both distributions underlined the susceptibility of the pH monitoring method to small changes in the setup (i.e. chip or position on the microscope). **b** Two droplet populations of pH 6 and pH 7.5 were generated separately and are distinguished by their fluorescence intensity distribution. When the populations were mixed and incubated together for 5 min, the pH started to equilibrate over the droplets exemplified by the intermediate fluorescence intensities. **c** pH equilibration proceeds until the populations were not distinguishable anymore. **d** pH decrease in a droplet population was specifically induced by adding perfluorinated oil in which a minute amount of acetic acid was dissolved

Very little is known about the behavior of pH mediating molecules in microfluidic emulsions with perfluorinated oils and surfactants. However, observations from Boitard et al. [7] suggest that protonated, organic acid molecules can diffuse out of the droplets. Therefore, we evaluated pH changes due to inter-droplet transport [8, 9] when incubating two distinct populations together. Each population contained unbuffered medium with 6-carboxyfluorescein at pH 6 or 7.5, respectively. Fluorescence intensity was measured droplet by droplet upon generation or reinjection (Fig. 2a). While droplets of the separated populations clearly present distinct fluorescent intensities, the intensities in the mixed population converge toward an intermediate value within seconds after mixing (2B). After 5 min, the populations in the mix can still be recognized in the histogram, albeit much closer than when separated. Further incubation together for 16 h resulted in completely undiscernible populations (Fig. 2c). This means that molecules and possibly even ions that change the pH can readily move between droplets.

### Employing inter-droplet transport for pH regulation

It has been recently shown that acidic compounds in the fluorinated oil are able to change the pH inside droplets [6, 10]. This, in line with our results on inter-droplet transport of pH molecules, can be exploited as a strategy to control the pH in an incubated droplet population. We show that acetic acid and diethylamine can be used to either decrease or increase pH in droplet populations. The measured fluorescence strongly changed as a result of spiking the recirculation oil with acetic acid or diethylamine both when measuring the average fluorescence value for a large sample of droplets in a microtiter plate, and when reinjecting and analyzing droplet by droplet (Fig. 2d). This confirms that these compounds can be used to externally control the pH inside of the incubated droplets.

### Model system for pH regulation during microbial droplet culture

**Fig. 3:**
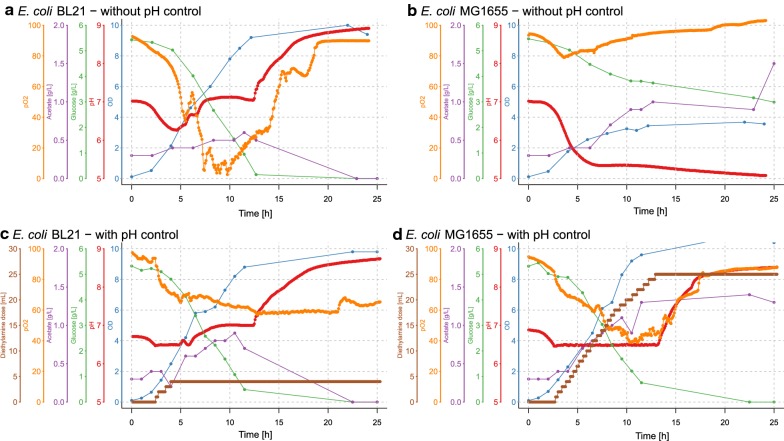
Lab-scale fermentations of the two model strains *E. coli* BL21 and MG1655 with and without pH control. Fermentations were carried out in 1 L bioreactors for 25 h, while DO, pH, temperature and stirring speed were measured continuously. Temperature and stirring speed were set to constant values (28 °C and 600 rpm respectively). OD_600_ and concentration of glucose and acetate were determined offline, for which samples of 10 mL were taken. Online and offline parameters are displayed for **a** *E. coli* BL21 without pH regulation, **b** *E. coli* MG1655 without pH regulation, **c** *E. coli* BL21 with pH regulation and **d** *E. coli* MG1655 with pH regulation. In case of pH regulation at pH 6.5, diethylamine was used as base

In order to test the applicability of droplet pH regulation, we tested the fermentation profiles of various *E. coli* strains, which are known to alter pH and are affected by it. When cultured in unbuffered media with glucose, the growth of *E. coli* MG1655 is truncated when the pH reaches 5.5 (Fig. 3b), presumably as a consequence of its own high production of acetate [11]. Contrarily, *E. coli* BL21 does not reach the same low pH due to a more restricted acetate production or simultaneous acetate consumption, as suggested by higher gene expression levels for the glyoxylate cycle [12] and the acetyl-CoA synthetase  [13]. Since acetate levels are kept stable at a low level of approximately 0.6 g/L, the pH does not fall below 6.4. Cell propagation continues until glucose is depleted (Fig. 3a). Then, cells additionally consume the secreted acetate and amino acids, which were likely dissimilated already in parallel to glucose before [13]. This results in further pH increase and more than twice the cell density. When performing the fermentation with pH control at a threshold at pH 6.5, the growth arrest of *E. coli* MG1655 is prevented even though acetate is still produced in high concentrations. By controlling the pH, *E. coli* MG1655 can consume the entire amount of glucose and reaches a similar optical density as *E. coli* BL21 (Fig. 3c, d).

**Fig. 4:**
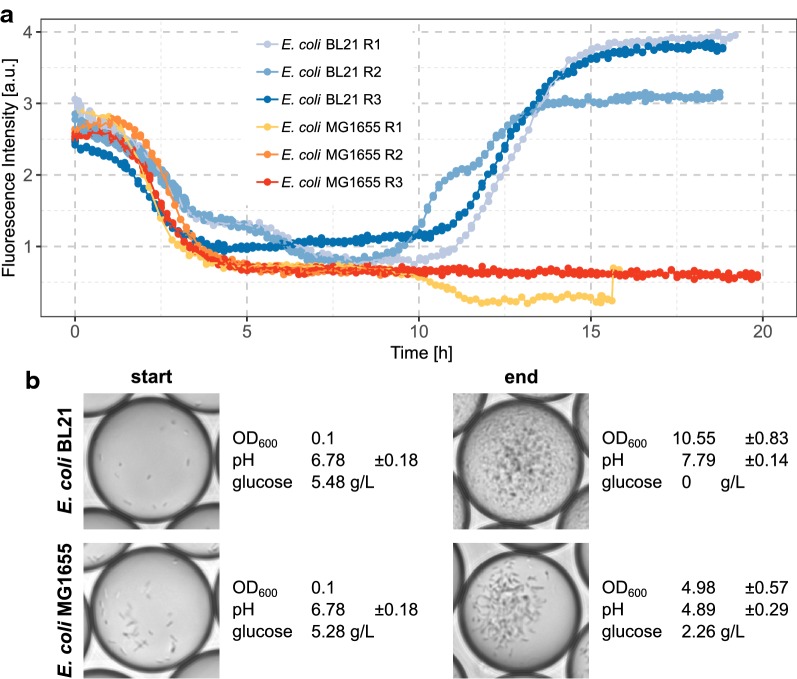
Development of fluorescence signal in dependency to pH changes in cultures of *E. coli* BL21 and *E. coli* MG1655 confined in microfluidic droplets without pH regulation. Droplet populations were incubated in customized incubators as part of the dynamic droplet incubation setup, with which oxygen was continuously supplied. The incubators were equipped with an excitation light source and a fiber guide leading to a photodiode. **a** Course of fluorescence intensity during microbial incubation in droplets for the replicate droplet cultivations with strains *E. coli* BL21 and *E. coli* MG1655. Fluorescence intensity was determined for entire droplet populations in the incubator. Rx indicates a different biological replicate from which the data was collected. **b** Representative images of droplets and offline parameters recorded for *E. coli* fermentation in pL droplets. Optical cell density (OD_600_), concentration of glucose and pH with standard pH electrode were measured before or after the incubation in droplets in the aqueous phase. Droplets were fused after incubation by using an anti-static gun [14]

The same tendency is observed when these strains are grown in microfluidic droplets incubated with the enhanced dynamic droplet incubation setup. The pH change is observed by following the green fluorescence intensity of the 6-carboxyfluorescein coencapsulated with the cells (4A). Based on measurements with a standard pH electrode before droplet generation, cultures for both strains start at a pH of approx. 6.5. For *E. coli* MG1655, the pH decreases within 4 h, similar to the observations in the bioreactor culture. *E. coli* BL21 also follows the same profile as in larger scaled culture, with an initial drop in pH and the subsequent increment of pH. Not only the development of the pH, but also the incomplete consumption of glucose by *E. coli* MG1655 and the reduced yield in biomass were reproduced in droplet cultivation (Fig. 4b). As a confirmation for the pH estimates obtained from fluorescence intensity monitoring, we also measured the pH with a pH electrode after droplet incubation and controlled emulsion breakage of the droplet population in the continous aqueous phase. As expected, the values are congruent to the expected trend and further strengthen our hypothesis regarding an equivalent bioprocess in pL droplets as in 1 L fermentations.

**Fig. 5:**
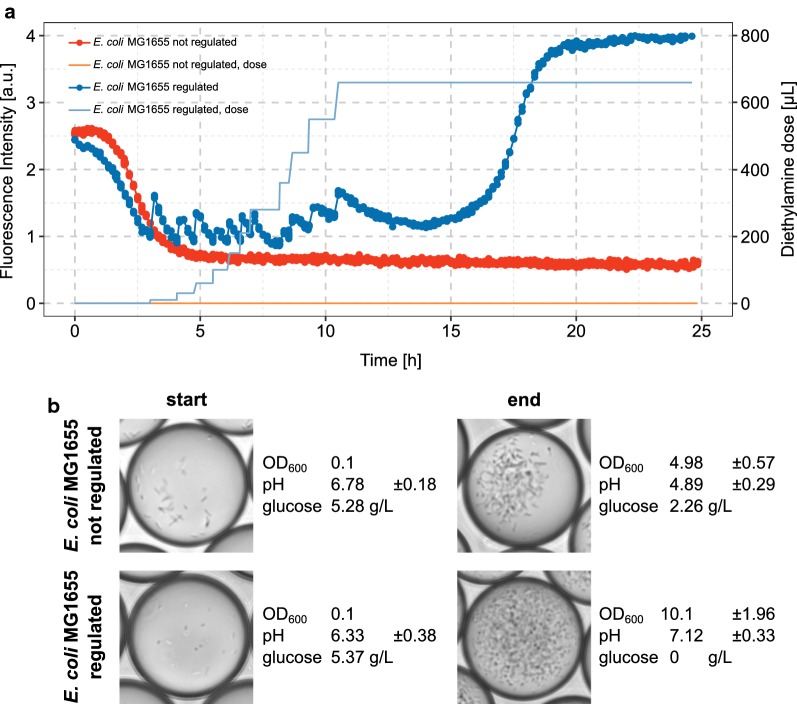
Development of fluorescent signal of *E. coli* MG1655 cultured in droplets under pH regulation. Like in experiments before, droplet populations were incubated in customized droplet incubators. Fluorescence of 6-carboxyfluorescein was monitored constantly as indication for pH development. When the fluorescence signal dropped below a value of 1, diethylamine dissolved in a ratio of 1:100 in the perfluorinated oil with surfactant at working concentration was administered by pipetting to the droplet population. **a** Fluorescence intensity over time for two droplet populations containing *E. coli* MG1655. One of them was treated with diethylamine and one served as untreated control. On the second y axis the cumulative amount of diethylamine treated oil added during the incubation is plotted. **b** Cell densities, glucose consumption and final pH measured with a pH electrode are compared for the regulated and not regulated droplet population

Adding minute amounts of diethylamine dissolved in oil to the continuous phase of droplet incubation via the oil reservoir of the recirculation setup results in homogeneous mixing of the modified oil with the droplet population and subsequent in control of the pH inside the droplets (Fig. 5a and Additional file 1: Figure S1). As expected, this enables *E. coli* MG1655 to grow further and reach similarly high cell densities as *E. coli* BL21. The complete consumption of glucose and the neutral pH is also in agreement with the higher biomass yield (Fig. 5b). Consequently, the observed bioprocess in pL droplets is equivalent to the course of the large-scale fermentation (Fig. 3). Also, when the regulation of pH is started at different time points during cultivation, the auto-recovery of neutral pH by *E. coli* MG1655 is restored (Additional file 1: Figure S1).

Here, continuous recirculation of the fluorinated oil ensures not only proper oxygenation, but serves as steerable carrier for the pH controlling molecules and a homogeneous mixing strategy. The addition of pH controlling acid or base can further be automated with a feedback loop according to the measurement provided by the fluorescence detector coupled to the droplet incubator.

## Conclusions

Here, we present a strategy that exploits inter-droplet transport to regulate pH inside picoliter droplets. We exemplify this strategy with the model organism *E. coli*, but it is applicable to all microbial cultivations in picoliter droplets. In most droplet microfluidic applications, populations consisting of thousands or millions of droplets are generated and incubated, from which particular variants should be distinguished in the context of a functional assay. However, cell propagation and reactions taking place in droplets are affected by pH changes even when those changes occur only in a fraction of the whole droplet population. Ignoring this issue leads to unreproducible and inconsistent results. Our strategy enables scientists to standardize conditions in droplets for physiological experimentation. Furthermore, new options in assay development can be exploited, since pH ranges can now be adjusted to select enzymes or microbes with activity at adverse or changing pH conditions. As most fluorescent dyes can be affected by changes in pH, it is also extremely useful to have the possibility to readjust the pH to values that allow optimal quantification in droplets. Controlled pH changes can also be used to trigger reactions in droplets [10] or other transport-mediated functionalities [8]. The possibility to regulate or rapidly change pH can also be crucial for chemical and nanoparticle synthesis applications in droplets [15].

The proposed strategy is a valuable feature for improved microbiological experimentation and screening assays in picoliter droplets. In fact, the possibility to control oxygen and pH reduces the experimental gap between microfluidic and larger scaled experimentation, such as performed in microtiter plates and bioreactors.

## Methods

### Droplet handling

Droplets were generated in a flow focusing unit on custom made PDMS chips by using syringe pumps (neMESYS, CETONI, Germany). Novec HFE7500 oil (3 M, Germany) with 0.5% surfactant (PicoSurf 1, Dolomite, UK) was used as perfluorinated, immiscible phase. The aqueous phase consisted of buffer or culture media with cells and 6-carboxyfluorescein. Droplets were collected and incubated in 3D printed incubators at 28 °C, [3] or in wells of microtiter plates at 500 rpm double orbital and 28 °C. Droplets were imaged immobile in chambers on PDMS chips with ×10 and ×40 magnification using a PCO edge camera (PCO AG, Germany).

### pH sensing

Bulk pH measurements for validation were performed with a conventional pH microelectrode (Mettler Toledo, Germany). For calibrations, pH was adjusted using 1 M NaOH (Roth, Germany) or 1 M HCl (Roth Germany).

6-carboxyfluorescein (Sigma-Aldrich, Germany), dissolved in 100% (v/v) DMSO, was diluted to a final concentration of 2 µg/mL in the aqueous phase. A 488 nm diode laser (LASOS Lasertechnik, Germany) was focused downstream of generation or reinjection structure on the microfluidic channel through which droplets flowed. Emitted light was collected by the optics of an inverse microscope (Axio Observer.Z1, Carl Zeiss, Germany) and detected by a photomultiplier module (H10721-20, Hamamatsu Photonics, Japan). For measurement of average pH in droplet population by 6-carboxyfluorescein, the entire droplet population was transferred to a standard black 96-well plate with clear bottom (Greiner Bio-One, Germany), which was sealed by a polyester film (Excel Scientific, Germany) to avoid evaporation. During longterm monitoring, the seals were perforated for oxygenation. Fluorescence intensity in plates was determined by a multimode plate reader (ClarioStar, BMG Labtech, Germany) at 486/8 nm excitation and 526/20 nm emission.

The dynamic droplet incubation setup was slightly modified (Additional file 1: Figure S2) by integrating holders for the excitation light source (Lumencor Spectra-X, USA) liquid light-guide and emitted light collection fiber (Pyroscience, Germany). Both are aligned toward the center of the droplet incubator, where droplets are always present and constantly recirculating. A laser clean up filter (AHF, Germany) was used to strongly restrict the excitation light to 488/4 nm. The collected emitted light was filtered with a 525/50 bandpass (Thorlabs) and detected with a photodiode. To minimize photobleaching, the light source was programmed to turn on for a second every 10 min.

### pH control in droplets

For increasing the pH in droplet populations, pure diethylamine (Sigma-Aldrich, Germany) was added to the perfluorinated oil with surfactant at working concentration in a ratio of 1:100. This conditioned oil was subsequently added stepwise to a droplet population in a ratio of 1:200.

For acidifying a droplet population, the perfluorinated oil was treated with acetic acid (Sigma-Aldrich, Germany) in the same way as described above.

### Culture conditions

*Escherichia coli* strains BL21 and MG1655 were stored at − 20 °C as cryo stocks containing 50% (v/v) of glycerol preservation medium. Cultivation was performed in unbuffered soy peptone yeast extract medium (SY) containing 5 g/L soy peptone (BD Difco, Germany), 5 g/L yeast extract (BD Difco, Germany) and 5 g/L NaCl (Roth, Germany) in aqua dest. Initial pH was adjusted to 6.75 before autoclaving for 20 min at 121 °C. Before usage, SY was complemented with 3 g/L sterile glucose (VWR international, Germany) for precultures or 5 g/L glucose for main cultures. Fresh medium was inoculated from first preculture to a start OD_600_ of 0.1 and incubated at 28 °C in glass Erlenmeyer flasks at 160 rpm until reaching mid exponential phase. Before droplet generation, cell suspensions were washed and diluted in SY medium + 5 g/L glucose to the starting cell concentration of OD_600_ 0.1.

### Culture in 1 L bioreactors

As described above, 2 precultures of both *E. coli* strains were produced to activate and synchronize the metabolism. 100 mL of the second preculture at OD_600_ 1 were used to inoculate 900 mL SY + 5 g/L glucose in Biostat B-DCU II bioreactors (Sartorius, Germany). The fermentations were carried out for 24 h at 28 °C with 600 rpm constant stirring and 0.5 L/min aeration. pH, pO2, temperature, air flow and stirring speed were continuously monitored online during the fermentation. For the offline determination of cell density, glucose and acetate concentration, a sample of 15 mL was taken under sterile conditions every 1.5 h. Glucose concentration was determined immediately with a YSI 2900 Biochemistry Analyzer (YSI Incorporated, USA) following the manufacturer protocol. Acetate concentration was determined by HPLC (Jasco, Germany) with an Aminex HPX-87H Ion Exclusion Column (Bio-Rad, USA), for which samples were stored at − 20 °C upon measurement. For pH regulation, 2 M diethylamine in aqua bidest was fed automatically into the fermenter through a pH control unit to prevent the pH from dropping below 6.5.

## Supplementary information

**Additional file 1: Figure S1.** Repetitions of *E. coli* MG1655 cultured in droplets under pH regulation. Droplet populations were incubated in customized droplet incubators. Fluorescence of 6-carboxyfluorescein was monitored constantly as indication for pH development. When the fluorescence signal dropped below a value of 1, diethylamine dissolved in a ratio of 1:100 in the perfluorinated oil with surfactant at working concentration was administered by pipetting to the droplet population. The fluorescence intensity is monitored over time for 3 replicates. Each droplet population contained *E. coli* MG1655 with the same starting cell density. Cell densities were measured at the end of the experiment after breaking the droplets, resulting in OD_600_ of 8.8, 7.63 and 7.8 respectively. The onset and course of the manual base dosage was chosen individually for each experiment. On the second y axis the cumulative amount of diethylamine treated oil added during the incubation is plotted. **Figure S2.** Dynamic droplet incubation with pH monitoring and control setup. Optical fibers are used to bring excitation light into the droplet incubator. Emitted light is collected with another fiber and the signal is measured in a photodiode. The oil reservoir is used to add the pH modifying molecules (diethylamine or acetic acid) already diluted in perfluorinated oil.

## Data Availability

All data generated or analyzed during this study are included in this published article. The datasets are available from the corresponding author on reasonable request.
