# Supplementary material for: Monitoring and external control of pH in microfluidic droplets during microbial culturing
Source: Microb Cell Fact. 2020 Jan 29;19:16. doi: 10.1186/s12934-020-1282-y (PMC6990587; doi:10.1186/s12934-020-1282-y)
Supplement: Supplementary file 1 — Additional file 1: Figure S1. Repetitions of E. coli MG1655 cultured in droplets under pH regulation. Droplet populations were incubated in customized droplet incubators. Fluorescence of 6-carboxyfluorescein was monitored constantly as indication for pH development. When the fluorescence signal dropped below a value of 1, diethylamine dissolved in a ratio of 1:100 in the perfluorinated oil with surfactant at working concentration was administered by pipetting to the droplet population. The fluorescence intensity is monitored over time for 3 replicates. Each droplet population contained E. coli MG1655 with the same starting cell density. Cell densities were measured at the end of the experiment after breaking the droplets, resulting in OD600 of 8.8, 7.63 and 7.8 respectively. The onset and course of the manual base dosage was chosen individually for each experiment. On the second y axis the cumulative amount of diethylamine treated oil added during the incubation is plotted. Figure S2. Dynamic droplet incubation with pH monitoring and control setup. Optical fibers are used to bring excitation light into the droplet incubator. Emitted light is collected with another fiber and the signal is measured in a photodiode. The oil reservoir is used to add the pH modifying molecules (diethylamine or acetic acid) already diluted in perfluorinated oil. [file 12934_2020_1282_MOESM1_ESM.docx]

Supplementary Information for:

Monitoring and external control of pH in microfluidic droplets during microbial culturing

Authors: Miguel Tovar*, Lisa Mahler*, Stefanie Buchheim, Martin Roth and Miriam A. Rosenbaum^#^

* contributed equally

^#^Correspondence: [miriam.rosenbaum@leibniz-hki.de](mailto:miriam.rosenbaum@leibniz-hki.de)

**Supplementary figures.**


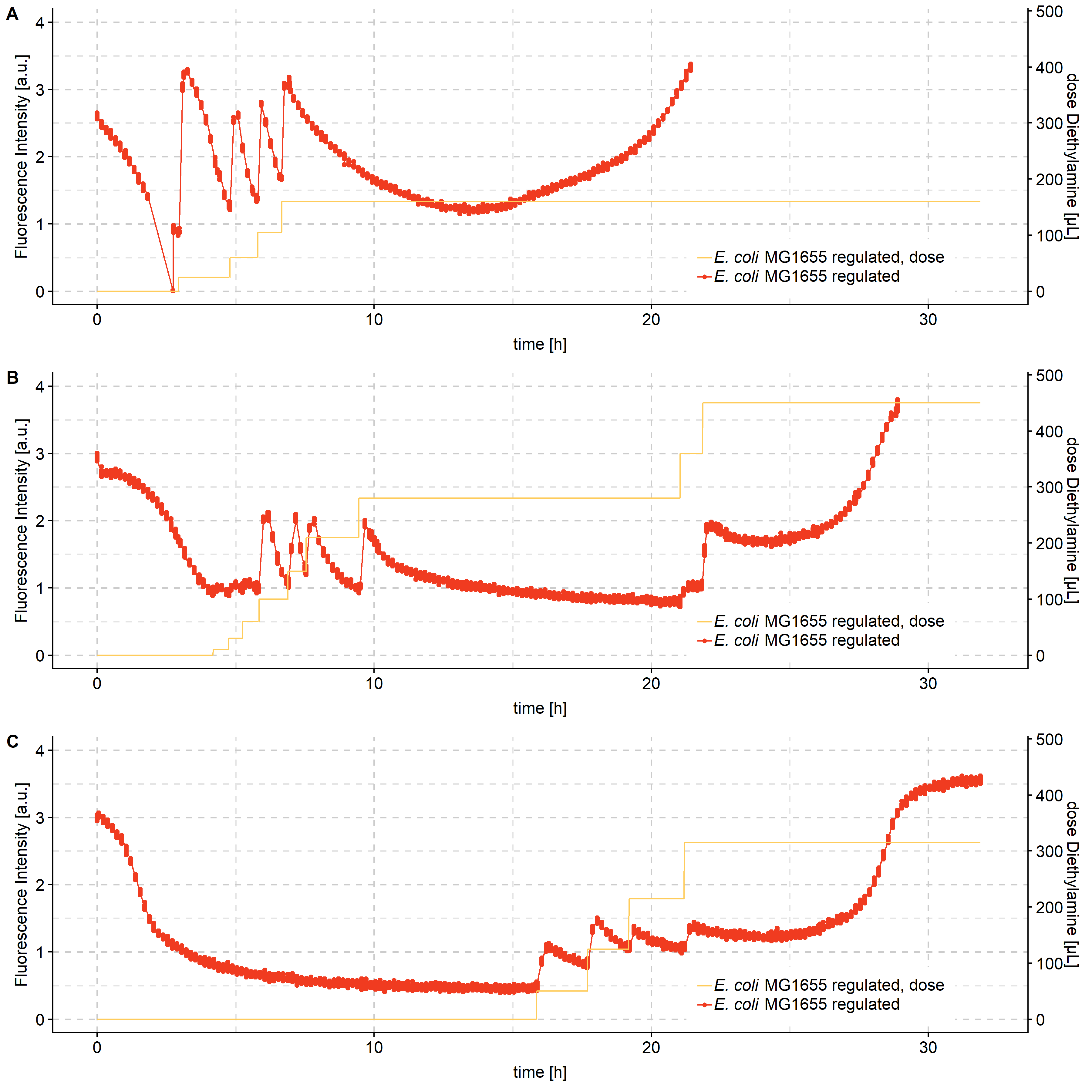


**Figure S1 – Repetitions of *E. coli* MG1655 cultured in droplets under pH regulation.** Droplet populations were incubated in customized droplet incubators. Fluorescence of 6-carboxyfluoresceine was monitored constantly as indication for pH development. When the fluorescence signal dropped below a value of 1, diethylamine dissolved in a ratio of 1:100 in the perfluorinated oil with surfactant at working concentration was administered by pipetting to the droplet population. The fluorescence intensity is monitored over time for 3 replicates. Each droplet population contained *E. coli* MG1655 with the same starting cell density. Cell densities were measured at the end of the experiment after breaking the droplets, resulting in OD_600_ of 8.8, 7.63 and 7.8 respectively. The onset and course of the manual base dosage was chosen individually for each experiment. On the second y axis the cumulative amount of diethylamine treated oil added during the incubation is plotted.


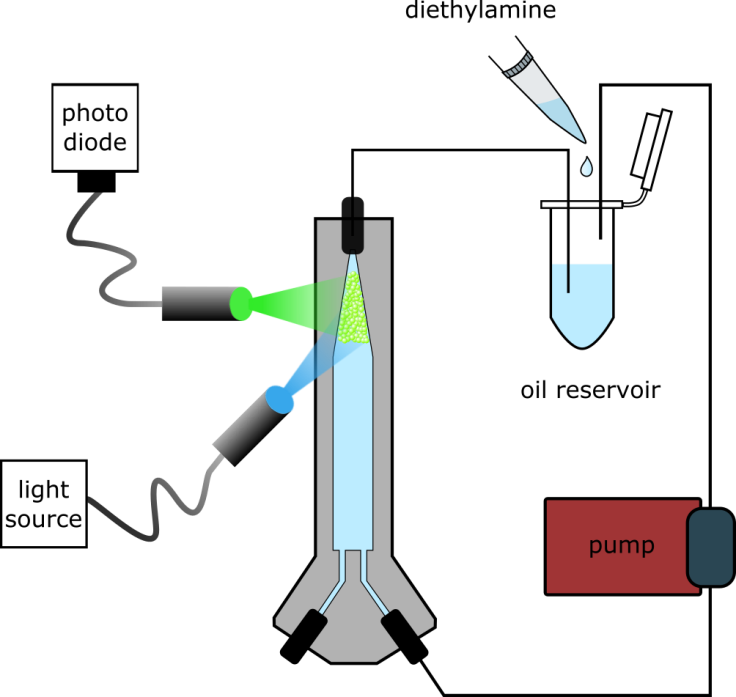


**Figure S2 – Dynamic droplet incubation with pH monitoring and control setup.** Optical fibers are used to bring excitation light into the droplet incubator. Emitted light is collected with another fiber and the signal is measured in a photodiode. The oil reservoir is used to add the pH modifying molecules (diethylamine or acetic acid) already diluted in perfluorinated oil.
